# Supplementary material for: Modeling glioblastoma heterogeneity as a dynamic network of cell states
Source: Mol Syst Biol. 2021 Sep 16;17(9):e10105. doi: 10.15252/msb.202010105 (PMC8444284; doi:10.15252/msb.202010105)
Supplement: Supplementary file 6 — Source Data for Figure 5 [file MSB-17-e10105-s004.zip › Figure5A_sourcedata/GSEA_3017/nosigdb_stateE.GseaPreranked.1622795031093/GAROFANO_IAVARONE_PPR_SUBTYPE.html]

Details for gene set GAROFANO\_IAVARONE\_PPR\_SUBTYPE[GSEA]

|  || Dataset | state63017 |
| Phenotype | NoPhenotypeAvailable |
| Upregulated in class | na\_neg |
| GeneSet | GAROFANO\_IAVARONE\_PPR\_SUBTYPE |
| Enrichment Score (ES) | -0.6097583 |
| Normalized Enrichment Score (NES) | -3.563595 |
| Nominal p-value | 0.0 |
| FDR q-value | 0.0 |
| FWER p-Value | 0.0 |
Table: GSEA Results Summary

  

Fig 1: Enrichment plot: GAROFANO\_IAVARONE\_PPR\_SUBTYPE      
 Profile of the Running ES Score & Positions of GeneSet Members on the Rank Ordered List

  

| PROBE | GENE SYMBOL | GENE\_TITLE | RANK IN GENE LIST | RANK METRIC SCORE | RUNNING ES | CORE ENRICHMENT || 1 | IDH1 |  |  | 218 | 0.284 | -0.3080 | No |
| 2 | LIN54 |  |  | 271 | -0.250 | -0.3802 | No |
| 3 | FIGNL1 |  |  | 290 | -0.257 | -0.4039 | No |
| 4 | DOT1L |  |  | 296 | -0.257 | -0.4092 | No |
| 5 | HNRNPAB |  |  | 312 | -0.261 | -0.4286 | No |
| 6 | HNRNPH1 |  |  | 334 | -0.265 | -0.4566 | No |
| 7 | MCM5 |  |  | 337 | -0.265 | -0.4575 | No |
| 8 | EPB41 |  |  | 345 | -0.268 | -0.4655 | No |
| 9 | KNTC1 |  |  | 352 | -0.269 | -0.4721 | No |
| 10 | HAUS3 |  |  | 358 | -0.271 | -0.4773 | No |
| 11 | ERI2 |  |  | 364 | -0.272 | -0.4824 | No |
| 12 | CENPP |  |  | 366 | -0.273 | -0.4819 | No |
| 13 | CDC25A |  |  | 368 | -0.273 | -0.4813 | No |
| 14 | POP7 |  |  | 370 | -0.273 | -0.4808 | No |
| 15 | PLEKHO1 |  |  | 385 | -0.277 | -0.4987 | No |
| 16 | HNRNPA2B1 |  |  | 391 | -0.279 | -0.5038 | No |
| 17 | DBF4B |  |  | 399 | -0.283 | -0.5117 | No |
| 18 | LSM4 |  |  | 406 | -0.284 | -0.5182 | No |
| 19 | SLIT1 |  |  | 408 | -0.285 | -0.5175 | No |
| 20 | NASP |  |  | 410 | -0.285 | -0.5169 | No |
| 21 | ZNF273 |  |  | 415 | -0.288 | -0.5205 | No |
| 22 | NIF3L1 |  |  | 418 | -0.289 | -0.5212 | No |
| 23 | CDK2 |  |  | 422 | -0.290 | -0.5234 | No |
| 24 | OIP5 |  |  | 426 | -0.291 | -0.5256 | No |
| 25 | FAM76B |  |  | 434 | -0.294 | -0.5334 | No |
| 26 | ZNF738 |  |  | 437 | -0.295 | -0.5341 | No |
| 27 | CTCF |  |  | 443 | -0.297 | -0.5391 | No |
| 28 | ZNF85 |  |  | 444 | -0.298 | -0.5369 | No |
| 29 | ZNF43 |  |  | 449 | -0.299 | -0.5404 | No |
| 30 | HNRNPD |  |  | 456 | -0.301 | -0.5468 | No |
| 31 | MCM4 |  |  | 457 | -0.301 | -0.5446 | No |
| 32 | TRIM28 |  |  | 460 | -0.304 | -0.5453 | No |
| 33 | PNRC2 |  |  | 461 | -0.304 | -0.5431 | No |
| 34 | ZNF530 |  |  | 464 | -0.304 | -0.5437 | No |
| 35 | DNMT1 |  |  | 466 | -0.304 | -0.5429 | No |
| 36 | ING2 |  |  | 468 | -0.305 | -0.5421 | No |
| 37 | SUV39H2 |  |  | 469 | -0.305 | -0.5399 | No |
| 38 | SMC1A |  |  | 472 | -0.307 | -0.5406 | No |
| 39 | NEIL3 |  |  | 475 | -0.307 | -0.5412 | No |
| 40 | EXOSC9 |  |  | 481 | -0.308 | -0.5461 | No |
| 41 | RECQL4 |  |  | 493 | -0.311 | -0.5595 | No |
| 42 | NUP205 |  |  | 495 | -0.313 | -0.5586 | No |
| 43 | RFC2 |  |  | 498 | -0.313 | -0.5592 | No |
| 44 | SRRT |  |  | 509 | -0.317 | -0.5711 | No |
| 45 | BARD1 |  |  | 510 | -0.318 | -0.5688 | No |
| 46 | NSMCE4A |  |  | 511 | -0.318 | -0.5665 | No |
| 47 | DNA2 |  |  | 513 | -0.318 | -0.5656 | No |
| 48 | CCDC150 |  |  | 516 | -0.319 | -0.5662 | No |
| 49 | HAUS1 |  |  | 518 | -0.320 | -0.5653 | No |
| 50 | POLA2 |  |  | 521 | -0.320 | -0.5658 | No |
| 51 | ZNF107 |  |  | 527 | -0.322 | -0.5706 | No |
| 52 | CEP135 |  |  | 532 | -0.325 | -0.5739 | No |
| 53 | PRIM1 |  |  | 539 | -0.328 | -0.5801 | No |
| 54 | ING1 |  |  | 541 | -0.329 | -0.5791 | No |
| 55 | DCLRE1C |  |  | 544 | -0.330 | -0.5796 | No |
| 56 | MSH2 |  |  | 548 | -0.332 | -0.5815 | No |
| 57 | CDC25C |  |  | 549 | -0.332 | -0.5791 | No |
| 58 | GPSM2 |  |  | 550 | -0.334 | -0.5766 | No |
| 59 | PLK4 |  |  | 551 | -0.334 | -0.5742 | No |
| 60 | RAD51 |  |  | 553 | -0.335 | -0.5732 | No |
| 61 | ZNF90 |  |  | 557 | -0.338 | -0.5750 | No |
| 62 | SAP30 |  |  | 558 | -0.339 | -0.5726 | No |
| 63 | TROAP |  |  | 562 | -0.341 | -0.5744 | No |
| 64 | SASS6 |  |  | 567 | -0.342 | -0.5776 | No |
| 65 | LSM5 |  |  | 568 | -0.342 | -0.5751 | No |
| 66 | PAXIP1 |  |  | 570 | -0.344 | -0.5741 | No |
| 67 | HNRNPA0 |  |  | 573 | -0.346 | -0.5744 | No |
| 68 | CKLF |  |  | 574 | -0.347 | -0.5719 | No |
| 69 | HMGB3 |  |  | 577 | -0.348 | -0.5722 | No |
| 70 | RCC1 |  |  | 582 | -0.350 | -0.5754 | No |
| 71 | GEN1 |  |  | 584 | -0.351 | -0.5743 | No |
| 72 | DSCC1 |  |  | 587 | -0.352 | -0.5746 | No |
| 73 | ZNF519 |  |  | 600 | -0.356 | -0.5890 | No |
| 74 | HIRIP3 |  |  | 603 | -0.359 | -0.5893 | No |
| 75 | ZWILCH |  |  | 604 | -0.360 | -0.5867 | No |
| 76 | FANCB |  |  | 606 | -0.361 | -0.5855 | No |
| 77 | POC1A |  |  | 611 | -0.363 | -0.5886 | No |
| 78 | KIF18B |  |  | 612 | -0.363 | -0.5859 | No |
| 79 | RFWD3 |  |  | 617 | -0.365 | -0.5890 | No |
| 80 | C18orf54 |  |  | 619 | -0.366 | -0.5878 | No |
| 81 | DDX11 |  |  | 622 | -0.370 | -0.5879 | No |
| 82 | WDR76 |  |  | 626 | -0.373 | -0.5895 | No |
| 83 | LCORL |  |  | 627 | -0.374 | -0.5868 | No |
| 84 | EXOSC8 |  |  | 630 | -0.376 | -0.5869 | No |
| 85 | RNASEH2A |  |  | 633 | -0.378 | -0.5870 | No |
| 86 | RMI1 |  |  | 637 | -0.381 | -0.5885 | No |
| 87 | ZNF704 |  |  | 649 | -0.392 | -0.6013 | No |
| 88 | POLE |  |  | 652 | -0.395 | -0.6013 | No |
| 89 | GINS2 |  |  | 658 | -0.399 | -0.6055 | No |
| 90 | SEPHS1 |  |  | 661 | -0.401 | -0.6055 | No |
| 91 | CDT1 |  |  | 665 | -0.406 | -0.6068 | Yes |
| 92 | MNS1 |  |  | 666 | -0.408 | -0.6039 | Yes |
| 93 | ARC |  |  | 667 | -0.408 | -0.6009 | Yes |
| 94 | SAE1 |  |  | 668 | -0.408 | -0.5980 | Yes |
| 95 | DSN1 |  |  | 671 | -0.410 | -0.5978 | Yes |
| 96 | KIF18A |  |  | 673 | -0.412 | -0.5963 | Yes |
| 97 | NUDT1 |  |  | 674 | -0.413 | -0.5933 | Yes |
| 98 | PKMYT1 |  |  | 681 | -0.420 | -0.5988 | Yes |
| 99 | RBMX |  |  | 682 | -0.421 | -0.5957 | Yes |
| 100 | DPF1 |  |  | 683 | -0.422 | -0.5927 | Yes |
| 101 | H2AFV |  |  | 684 | -0.423 | -0.5896 | Yes |
| 102 | CENPO |  |  | 686 | -0.424 | -0.5880 | Yes |
| 103 | FANCA |  |  | 691 | -0.425 | -0.5906 | Yes |
| 104 | STIL |  |  | 692 | -0.425 | -0.5875 | Yes |
| 105 | CKAP5 |  |  | 694 | -0.426 | -0.5859 | Yes |
| 106 | RANBP1 |  |  | 697 | -0.427 | -0.5856 | Yes |
| 107 | CHEK1 |  |  | 704 | -0.433 | -0.5910 | Yes |
| 108 | TIMELESS |  |  | 707 | -0.435 | -0.5907 | Yes |
| 109 | POLD3 |  |  | 710 | -0.437 | -0.5904 | Yes |
| 110 | ZNF93 |  |  | 712 | -0.438 | -0.5887 | Yes |
| 111 | LMNB2 |  |  | 713 | -0.438 | -0.5855 | Yes |
| 112 | SKA1 |  |  | 719 | -0.447 | -0.5894 | Yes |
| 113 | MASTL |  |  | 721 | -0.451 | -0.5875 | Yes |
| 114 | POLQ |  |  | 726 | -0.455 | -0.5899 | Yes |
| 115 | SPAG5 |  |  | 729 | -0.458 | -0.5894 | Yes |
| 116 | GINS1 |  |  | 730 | -0.459 | -0.5861 | Yes |
| 117 | CCNF |  |  | 732 | -0.460 | -0.5842 | Yes |
| 118 | POLA1 |  |  | 733 | -0.460 | -0.5809 | Yes |
| 119 | BRIP1 |  |  | 734 | -0.461 | -0.5775 | Yes |
| 120 | LBR |  |  | 739 | -0.463 | -0.5799 | Yes |
| 121 | E2F8 |  |  | 741 | -0.467 | -0.5779 | Yes |
| 122 | SMC3 |  |  | 743 | -0.470 | -0.5759 | Yes |
| 123 | NUCKS1 |  |  | 744 | -0.471 | -0.5725 | Yes |
| 124 | PTMA |  |  | 745 | -0.475 | -0.5691 | Yes |
| 125 | TK1 |  |  | 747 | -0.480 | -0.5671 | Yes |
| 126 | LIG1 |  |  | 752 | -0.485 | -0.5692 | Yes |
| 127 | WDR62 |  |  | 753 | -0.486 | -0.5657 | Yes |
| 128 | PCDH10 |  |  | 757 | -0.491 | -0.5664 | Yes |
| 129 | TRIP13 |  |  | 760 | -0.498 | -0.5657 | Yes |
| 130 | SOX11 |  |  | 764 | -0.504 | -0.5663 | Yes |
| 131 | GMNN |  |  | 766 | -0.510 | -0.5640 | Yes |
| 132 | RTKN2 |  |  | 767 | -0.511 | -0.5603 | Yes |
| 133 | CDC45 |  |  | 773 | -0.521 | -0.5637 | Yes |
| 134 | DTYMK |  |  | 774 | -0.522 | -0.5599 | Yes |
| 135 | STMN1 |  |  | 778 | -0.529 | -0.5603 | Yes |
| 136 | DEPDC1B |  |  | 779 | -0.533 | -0.5565 | Yes |
| 137 | CENPA |  |  | 782 | -0.538 | -0.5554 | Yes |
| 138 | SKA3 |  |  | 783 | -0.539 | -0.5515 | Yes |
| 139 | CEP152 |  |  | 785 | -0.542 | -0.5490 | Yes |
| 140 | BLM |  |  | 786 | -0.543 | -0.5451 | Yes |
| 141 | FANCD2 |  |  | 787 | -0.544 | -0.5412 | Yes |
| 142 | INCENP |  |  | 789 | -0.558 | -0.5385 | Yes |
| 143 | NCAPD3 |  |  | 790 | -0.559 | -0.5345 | Yes |
| 144 | ATAD5 |  |  | 791 | -0.559 | -0.5305 | Yes |
| 145 | HAUS8 |  |  | 792 | -0.560 | -0.5264 | Yes |
| 146 | HMGN2 |  |  | 795 | -0.564 | -0.5252 | Yes |
| 147 | RACGAP1 |  |  | 796 | -0.566 | -0.5211 | Yes |
| 148 | HMGB1 |  |  | 797 | -0.567 | -0.5170 | Yes |
| 149 | ASF1B |  |  | 798 | -0.568 | -0.5128 | Yes |
| 150 | CENPJ |  |  | 799 | -0.568 | -0.5087 | Yes |
| 151 | RFC3 |  |  | 801 | -0.569 | -0.5060 | Yes |
| 152 | CEP78 |  |  | 803 | -0.570 | -0.5033 | Yes |
| 153 | CENPN |  |  | 805 | -0.574 | -0.5006 | Yes |
| 154 | CDC6 |  |  | 807 | -0.584 | -0.4978 | Yes |
| 155 | CDCA8 |  |  | 808 | -0.585 | -0.4936 | Yes |
| 156 | PLK1 |  |  | 809 | -0.586 | -0.4893 | Yes |
| 157 | CHAF1A |  |  | 810 | -0.588 | -0.4851 | Yes |
| 158 | FOXM1 |  |  | 814 | -0.593 | -0.4850 | Yes |
| 159 | CDCA2 |  |  | 816 | -0.597 | -0.4821 | Yes |
| 160 | MCM7 |  |  | 817 | -0.599 | -0.4778 | Yes |
| 161 | LMNB1 |  |  | 818 | -0.606 | -0.4734 | Yes |
| 162 | MND1 |  |  | 821 | -0.615 | -0.4718 | Yes |
| 163 | PIF1 |  |  | 822 | -0.616 | -0.4674 | Yes |
| 164 | CKAP2L |  |  | 826 | -0.625 | -0.4671 | Yes |
| 165 | DNAJC9 |  |  | 828 | -0.633 | -0.4639 | Yes |
| 166 | AURKB |  |  | 829 | -0.634 | -0.4594 | Yes |
| 167 | HELLS |  |  | 830 | -0.635 | -0.4548 | Yes |
| 168 | DTL |  |  | 831 | -0.637 | -0.4501 | Yes |
| 169 | PCNA |  |  | 832 | -0.640 | -0.4455 | Yes |
| 170 | CENPW |  |  | 835 | -0.643 | -0.4437 | Yes |
| 171 | BUB1 |  |  | 837 | -0.653 | -0.4404 | Yes |
| 172 | ANP32E |  |  | 841 | -0.676 | -0.4398 | Yes |
| 173 | KIF22 |  |  | 842 | -0.678 | -0.4349 | Yes |
| 174 | BIRC5 |  |  | 843 | -0.679 | -0.4300 | Yes |
| 175 | CENPM |  |  | 844 | -0.682 | -0.4250 | Yes |
| 176 | NCAPH |  |  | 845 | -0.685 | -0.4201 | Yes |
| 177 | BRCA1 |  |  | 846 | -0.691 | -0.4151 | Yes |
| 178 | ZWINT |  |  | 847 | -0.692 | -0.4101 | Yes |
| 179 | SPC24 |  |  | 848 | -0.694 | -0.4050 | Yes |
| 180 | CENPH |  |  | 849 | -0.694 | -0.4000 | Yes |
| 181 | BRCA2 |  |  | 850 | -0.694 | -0.3950 | Yes |
| 182 | ECT2 |  |  | 854 | -0.717 | -0.3941 | Yes |
| 183 | KIF2C |  |  | 855 | -0.721 | -0.3889 | Yes |
| 184 | NUF2 |  |  | 856 | -0.721 | -0.3836 | Yes |
| 185 | CENPK |  |  | 857 | -0.722 | -0.3784 | Yes |
| 186 | KIF4A |  |  | 861 | -0.736 | -0.3773 | Yes |
| 187 | FEN1 |  |  | 862 | -0.736 | -0.3720 | Yes |
| 188 | CCNB2 |  |  | 865 | -0.745 | -0.3695 | Yes |
| 189 | RRM1 |  |  | 866 | -0.749 | -0.3641 | Yes |
| 190 | ARHGAP11A |  |  | 867 | -0.756 | -0.3586 | Yes |
| 191 | NDC80 |  |  | 869 | -0.762 | -0.3545 | Yes |
| 192 | BUB1B |  |  | 871 | -0.766 | -0.3504 | Yes |
| 193 | CCNA2 |  |  | 872 | -0.770 | -0.3448 | Yes |
| 194 | KIF23 |  |  | 873 | -0.772 | -0.3392 | Yes |
| 195 | MELK |  |  | 874 | -0.776 | -0.3336 | Yes |
| 196 | TMPO |  |  | 875 | -0.778 | -0.3280 | Yes |
| 197 | CKAP2 |  |  | 878 | -0.781 | -0.3252 | Yes |
| 198 | NCAPG2 |  |  | 879 | -0.781 | -0.3195 | Yes |
| 199 | MCM10 |  |  | 881 | -0.798 | -0.3151 | Yes |
| 200 | H2AFX |  |  | 882 | -0.799 | -0.3094 | Yes |
| 201 | KIFC1 |  |  | 883 | -0.807 | -0.3035 | Yes |
| 202 | KIF14 |  |  | 885 | -0.809 | -0.2991 | Yes |
| 203 | EZH2 |  |  | 886 | -0.810 | -0.2932 | Yes |
| 204 | UBE2S |  |  | 887 | -0.815 | -0.2873 | Yes |
| 205 | FAM111B |  |  | 888 | -0.822 | -0.2814 | Yes |
| 206 | KIF15 |  |  | 890 | -0.834 | -0.2768 | Yes |
| 207 | CDCA5 |  |  | 892 | -0.841 | -0.2721 | Yes |
| 208 | H2AFZ |  |  | 893 | -0.849 | -0.2659 | Yes |
| 209 | RAD51AP1 |  |  | 894 | -0.853 | -0.2598 | Yes |
| 210 | KPNA2 |  |  | 895 | -0.858 | -0.2536 | Yes |
| 211 | DBF4 |  |  | 896 | -0.864 | -0.2473 | Yes |
| 212 | SMC2 |  |  | 897 | -0.867 | -0.2410 | Yes |
| 213 | CENPE |  |  | 899 | -0.873 | -0.2361 | Yes |
| 214 | FANCI |  |  | 901 | -0.882 | -0.2312 | Yes |
| 215 | SMC4 |  |  | 902 | -0.886 | -0.2248 | Yes |
| 216 | DUT |  |  | 903 | -0.887 | -0.2183 | Yes |
| 217 | XRCC2 |  |  | 908 | -0.907 | -0.2175 | Yes |
| 218 | CKS1B |  |  | 909 | -0.912 | -0.2109 | Yes |
| 219 | HJURP |  |  | 910 | -0.921 | -0.2042 | Yes |
| 220 | GTSE1 |  |  | 912 | -0.941 | -0.1988 | Yes |
| 221 | KIF11 |  |  | 913 | -0.957 | -0.1919 | Yes |
| 222 | MYBL2 |  |  | 914 | -0.959 | -0.1849 | Yes |
| 223 | CLSPN |  |  | 916 | -0.964 | -0.1794 | Yes |
| 224 | TYMS |  |  | 917 | -0.965 | -0.1724 | Yes |
| 225 | PRC1 |  |  | 919 | -0.980 | -0.1667 | Yes |
| 226 | MAD2L1 |  |  | 921 | -0.990 | -0.1610 | Yes |
| 227 | UBE2T |  |  | 922 | -0.994 | -0.1538 | Yes |
| 228 | SPC25 |  |  | 924 | -1.033 | -0.1477 | Yes |
| 229 | ESCO2 |  |  | 926 | -1.067 | -0.1414 | Yes |
| 230 | UBE2C |  |  | 928 | -1.108 | -0.1348 | Yes |
| 231 | NUSAP1 |  |  | 929 | -1.112 | -0.1268 | Yes |
| 232 | TPX2 |  |  | 930 | -1.124 | -0.1187 | Yes |
| 233 | NCAPG |  |  | 931 | -1.142 | -0.1104 | Yes |
| 234 | ASPM |  |  | 932 | -1.146 | -0.1021 | Yes |
| 235 | ATAD2 |  |  | 933 | -1.162 | -0.0937 | Yes |
| 236 | HIST2H2AC |  |  | 934 | -1.162 | -0.0853 | Yes |
| 237 | CENPF |  |  | 935 | -1.200 | -0.0766 | Yes |
| 238 | HMGB2 |  |  | 937 | -1.238 | -0.0691 | Yes |
| 239 | CDK1 |  |  | 938 | -1.248 | -0.0600 | Yes |
| 240 | RRM2 |  |  | 940 | -1.403 | -0.0513 | Yes |
| 241 | MKI67 |  |  | 941 | -1.407 | -0.0411 | Yes |
| 242 | TOP2A |  |  | 942 | -1.417 | -0.0309 | Yes |
| 243 | HIST1H1B |  |  | 945 | -1.903 | -0.0199 | Yes |
| 244 | HIST1H4C |  |  | 946 | -2.752 | -0.0000 | Yes |
Table: GSEA details [plain text format]

  

Fig 2: GAROFANO\_IAVARONE\_PPR\_SUBTYPE: Random ES distribution      
 Gene set null distribution of ES for **GAROFANO\_IAVARONE\_PPR\_SUBTYPE**
